# SARS-CoV-2: Current Tools to Fight COVID-19 ST-Elevation Myocardial Infarction

**DOI:** 10.7759/cureus.43539

**Published:** 2023-08-15

**Authors:** Syed Ifthikar, Javad Savoj, Harjeet Singh, Patrick Hu

**Affiliations:** 1 Cardiology, HCA Healthcare Riverside, Riverside, USA; 2 Internal Medicine, HCA Healthcare Riverside, Riverside, USA; 3 Interventional Cardiology, HCA Healthcare Riverside, Riverside, USA

**Keywords:** thrombolytic, impella device, percutaneous cardiac intervention, coronary stent, balloon pump, thrombus, myocardial infarction, mechanical thrombectomy (mt), covid 19, st-elevation myocardial infarction (stemi)

## Abstract

The capacity of the severe acute respiratory syndrome coronavirus 2 (SARS-CoV-2) to wreak havoc on the inflammatory and coagulation pathways via the cytokine storm has led to over 6.3 million fatalities globally. Based on recent data, the mechanism predominately involves the formation of microvascular thrombosis when pertaining to cardiovascular disease. However, a subset of coronavirus disease-2019 (COVID-19)-positive patients present emergently with acute ST-elevation myocardial infarction (STEMI) are found to have severe epicardial thrombosis which is refractory to traditional coronary revascularization. We have noted mortality in these patients presenting to our facility to be as high as 90% and all angiographically confirmed to have thrombus which was refractory to traditional therapy. We present a case series of COVID-19-positive patients presenting with STEMI found to have epicardial thrombus who were treated with different traditional STEMI therapies but with fatal outcomes. Other possible techniques including mechanical thrombectomy, optimizing traditional and nontraditional anticoagulation therapy with the use of early hemodynamic support may prove more efficacious to destroy thrombus and potentially improve mortality.

## Introduction

As of June 2021, the coronavirus disease-2019 (COVID-19) pandemic had affected more than 534 million people worldwide, leading to more than 6.3 million deaths. One of the devastating components of severe acute respiratory syndrome coronavirus 2 (SARS-CoV-2) is the development of the cytokine storm in which the immune system is hijacked and causes havoc on both the inflammatory and coagulation pathways [1].

Extensive investigation of the virus based on autopsies initially revealed microvascular thrombosis concerning cardiovascular disease [2]. However, a subset of COVID-19 patients present emergently with acute ST-elevation myocardial infarction (STEMI) and severe epicardial thrombosis, which is refractory to traditional coronary revascularization. This is despite adjunctive aggressive mechanical and pharmacological therapies such as balloon angioplasty, aspiration thrombectomy, P2Y12 inhibitors, and glycoprotein-2b/3a inhibitors.

In our single STEMI receiving center, mortality in our COVID-19 patients presenting with acute ST elevation was as high as 90%, and all patients were angiographically confirmed to have thrombus refractory to traditional therapy. We present a case series of COVID-19 patients with STEMI and concurrent epicardial thrombus. These patients were treated with different traditional STEMI therapies. We also review possible techniques that may prove more effective in destroying thrombus and potentially improving mortality.

## Case presentation

Case 1

**Figure 1:**
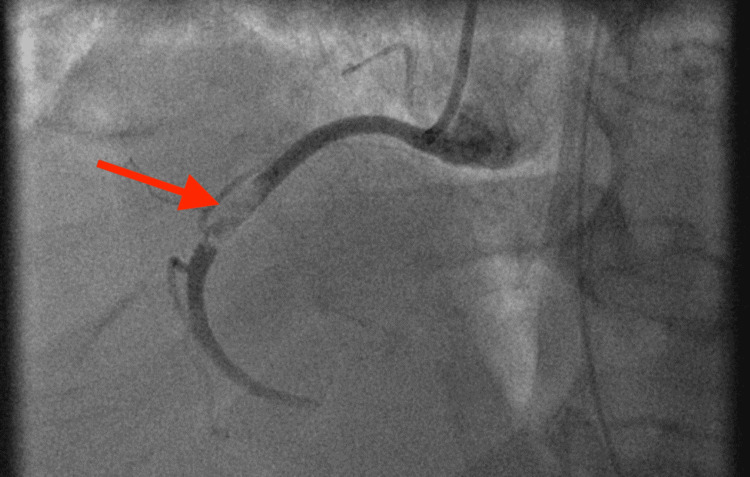
Initial coronary angiography revealed mid-RCA occlusion (arrow). RCA, right coronary artery.

This patient was a 65-year-old man with newly diagnosed diabetes who initially presented flu-like symptoms and was positive for COVID-19. On day seven of hospitalization, the patient became hypoxic and confused and was consequently intubated. An electrocardiogram (ECG) revealed a third-degree atrioventricular (AV) block and inferior lead ST elevation with reciprocal changes. The patient was taken immediately to the catheterization lab. He was found to have a thrombus in the mid-right coronary artery (RCA; Figure 1).

**Figure 2:**
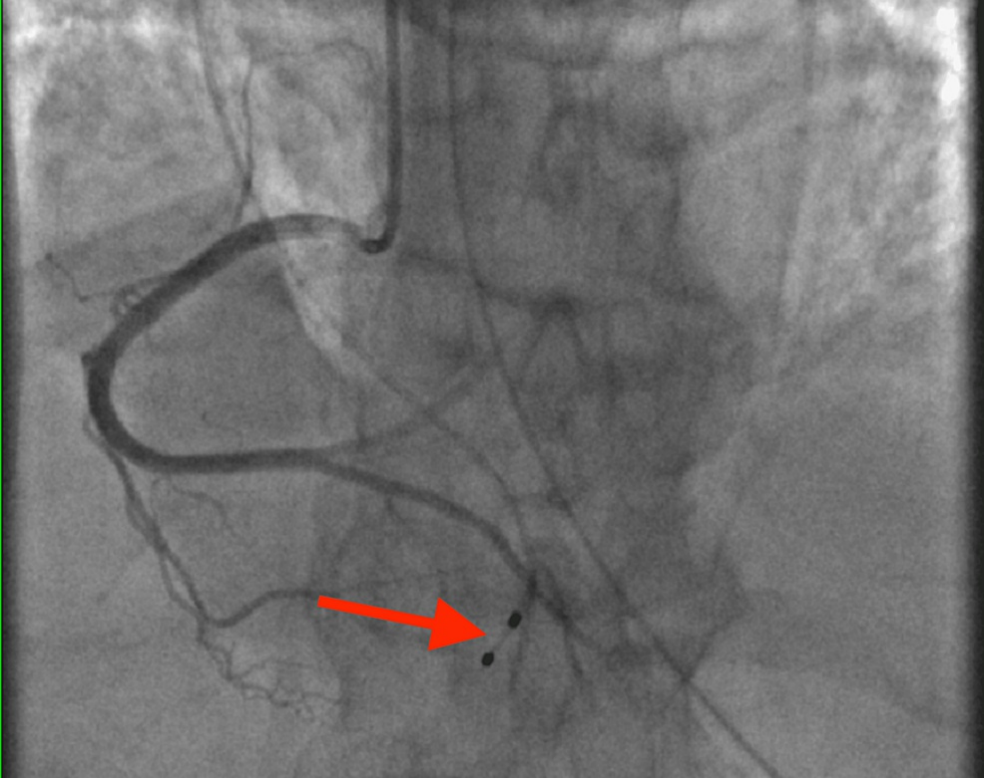
After unsuccessful thrombolysis with bivalirudin and tirofiban was attempted, a coronary stent at the mid-RCA occlusion was placed (arrow). This resulted in increased grade 2 coronary flow. RCA, right coronary artery.

After placing a temporary pacemaker and unsuccessful aspiration thrombectomy, the proximal RCA was stented (Figure 2) with resulting thrombolysis in myocardial infarction grade two flow. Bivalirudin and tirofiban were used during the procedure. The left ventriculogram showed a severely depressed left ventricular ejection fraction (LVEF) of 20% to 30%. Despite receiving dual antiplatelet therapy with aspirin and ticagrelor, as well as a heparin drip after the procedure for the rest of his hospitalization, the patient was found to have a new-onset deep vein thrombosis in the lower extremities three days after the cardiac catheterization. He died several days later. 

Case 2

**Figure 3:**
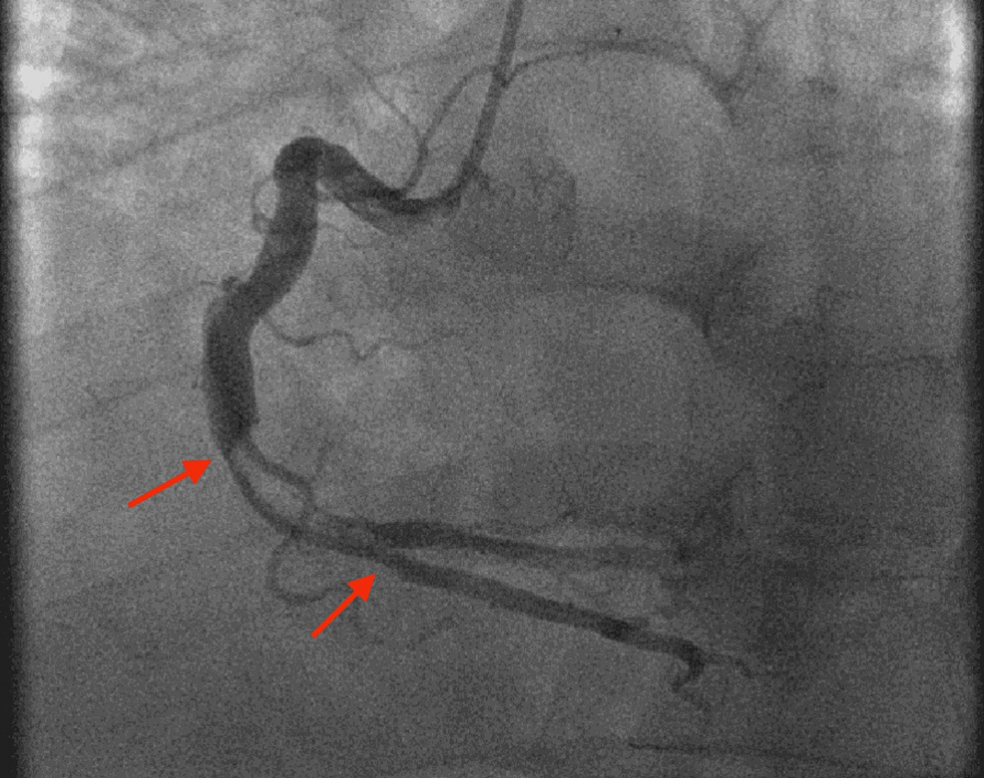
Coronary angiography revealed large thrombi in the proximal and mid-RCA (arrows) that were resistant to mechanical thrombectomy as well as systemic pharmacologic thrombolysis. RCA, right coronary artery.

This patient was a 50-year-old man with a history of hypertension who had presented to a hospital with shortness of breath and was diagnosed with COVID-19 pneumonia. On day four, he began to have chest pain. His ECG showed inferior and anterolateral ST elevations. He was transferred emergently to a STEMI receiving center. Upon arrival, he was in respiratory distress and needed bilevel-positive airway pressure for oxygen support. His coronary angiography revealed large thrombi in the proximal and mid-RCA (Figure 3). Despite multiple attempts with coronary and even peripheral thrombectomy devices, mechanical thrombectomy was unsuccessful. The patient was also started on a tirofiban and a heparin drip. An echocardiogram showed his LVEF was 55%. Despite aggressive treatment, he developed acute respiratory distress syndrome and bilateral pneumothorax and died five days later. 

Case 3

**Figure 4:**
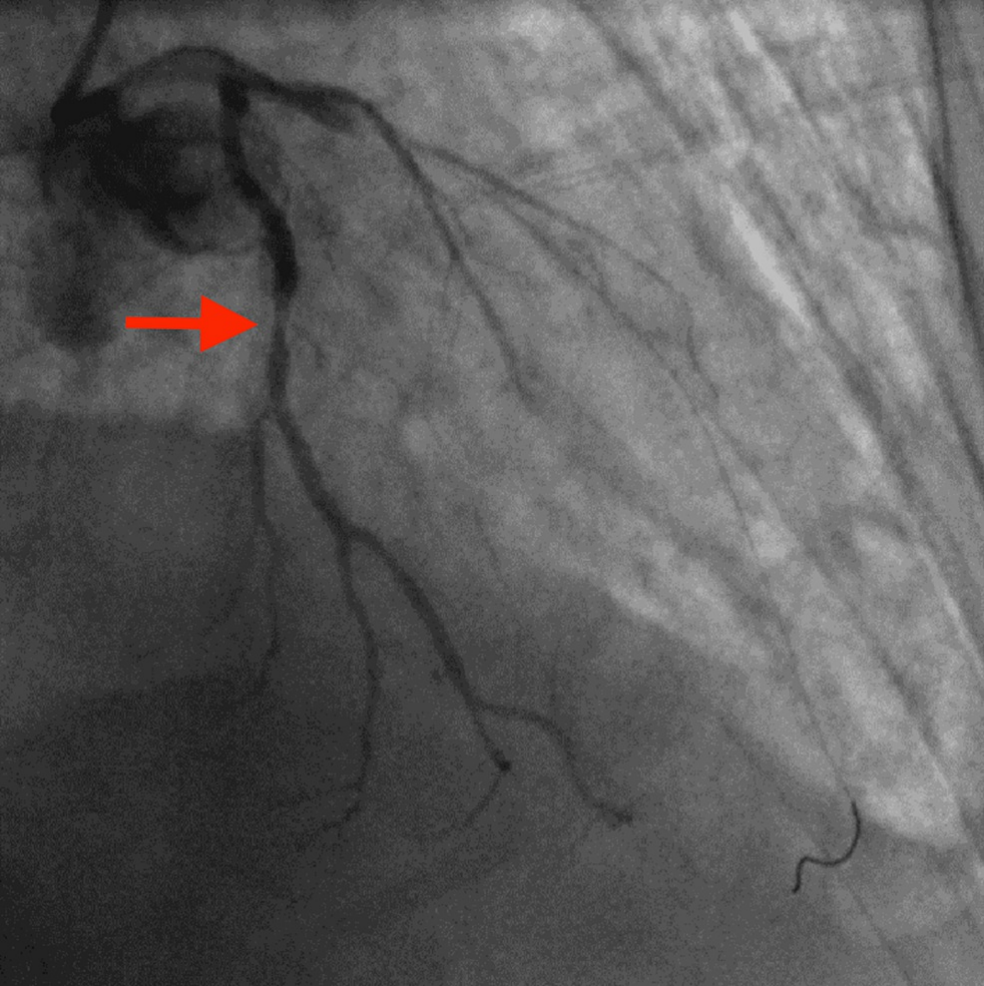
Coronary angiogram showed acute diffuse subtotal thrombotic occlusion of the LAD (arrow). Furthermore, there was severe multivessel coronary artery disease, including the proximal and mid segments of the left circumflex artery and the mid-segment of the RCA (not shown in the image). LAD, left anterior descending artery; RCA, right coronary artery.

The patient was a 70-year-old man with a history of uncontrolled diabetes, hypertension, hyperlipidemia, chronic obstructive pulmonary disease, and COVID-19 who presented from a skilled nursing facility with acute anterior STEMI. The emergency coronary angiogram showed acute diffuse subtotal thrombotic occlusion of the left anterior descending artery (LAD; Figure 4). He also had severe multivessel coronary artery disease, including the proximal and mid segments of the left circumflex artery and the mid-segment of the RCA. Multiple attempts at balloon angioplasty of the LAD thrombus failed to establish reperfusion. Tirofiban and bivalirudin drips were started at the beginning of the procedure.

**Figure 5:**
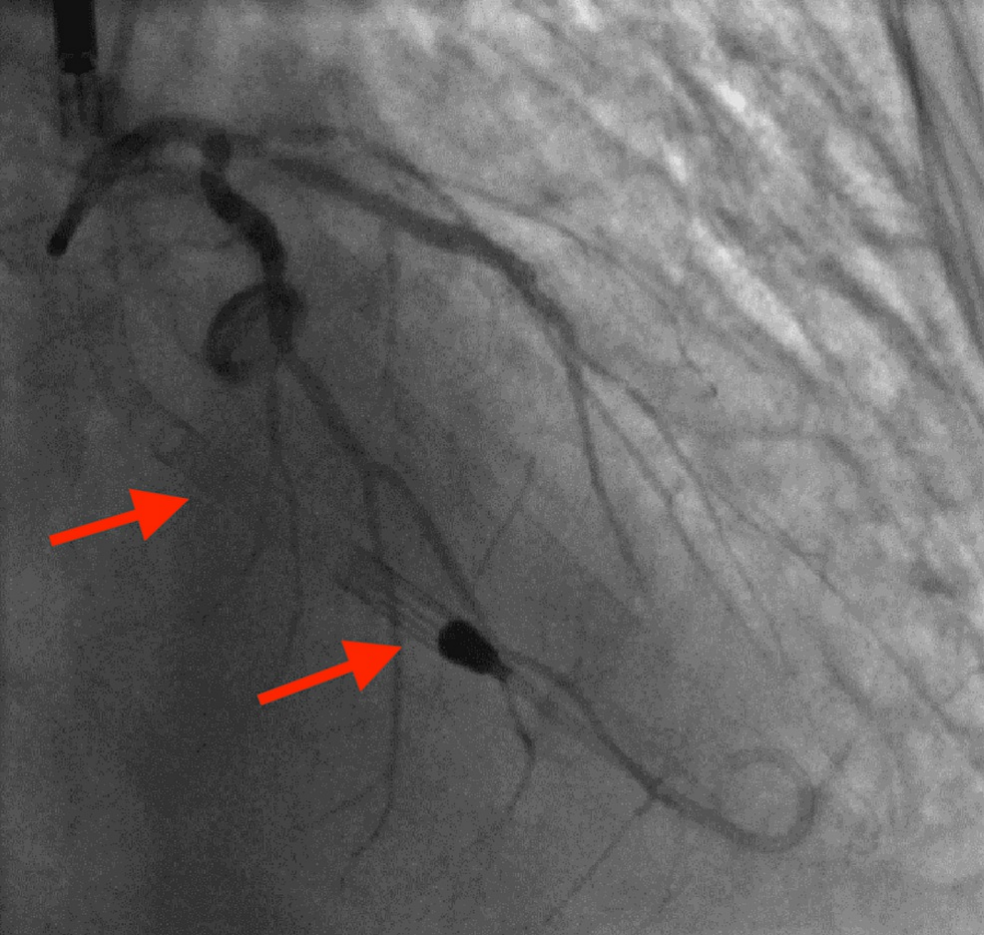
Following the administration of intracoronary alteplase injection, the patient became hypotensive, and an Impella device was placed for cardiopulmonary resuscitation efforts (arrows). Impella is a type of mechanical circulatory support device.

After an unsuccessful aspiration thrombectomy, a decision was made to proceed with intracoronary recombinant thrombolytic with alteplase 6 mg. The care team initiated cardiopulmonary resuscitation when the patient became hypotensive and was in cardiac arrest. After spontaneous circulation was returned, an Impella percutaneous circulatory support device was placed (Figure 5). After alteplase administration, a repeated angiogram showed partial resolution of coronary thrombosis in the LAD. A drug-eluting stent was successfully placed across the LAD, and the patient was transferred to the cardiac intensive care unit. However, two days later, the patient developed multiorgan failure and refractory pulseless electrical activity/cardiac arrest, from which he could not be resuscitated. 

Case 4

**Figure 6:**
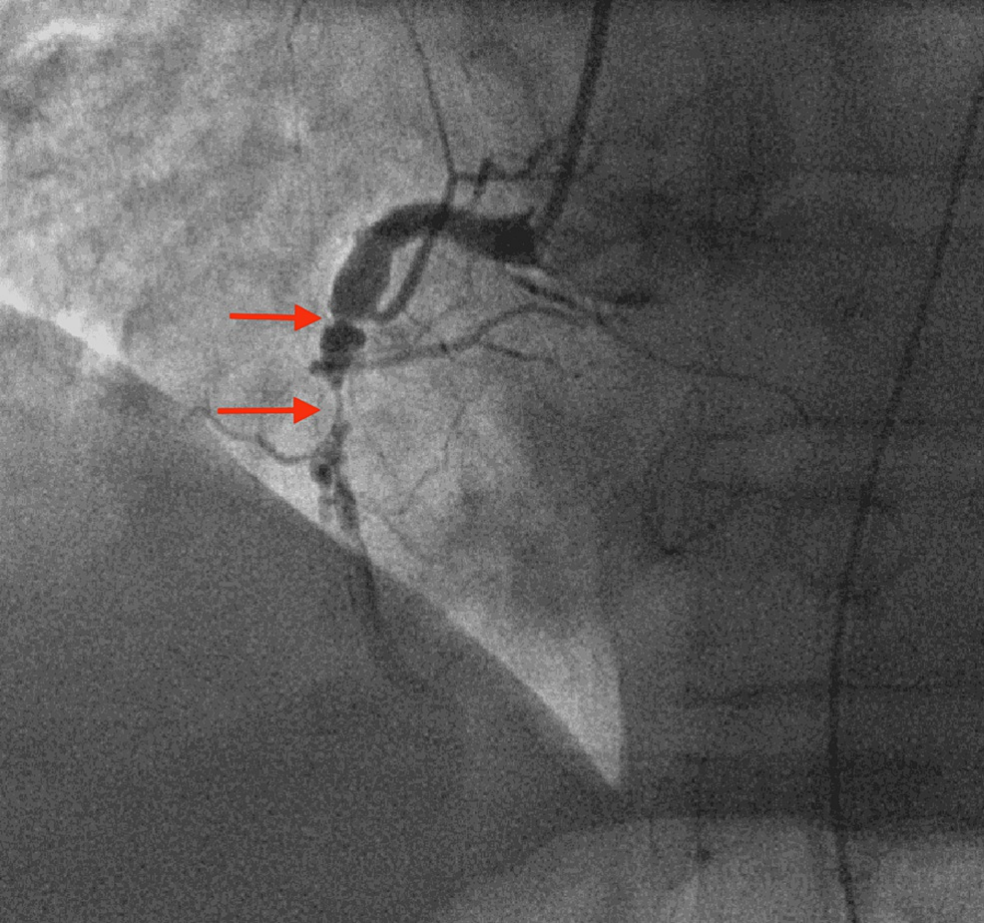
Coronary angiography revealed subtotal RCA lesion (arrows) with left-to-right collaterals. RCA, right coronary artery.

This patient was a 77-year-old man with a history of hypertension who presented with fever, chest pain, and worsening shortness of breath. Upon arrival, the patient was hypoxic, and his ECG showed STEMI in the inferior leads. The patient was intubated for increased work of breathing due to hypoxia. His chest radiograph was consistent with diffuse bilateral pneumonia, and the COVID-19 test was positive. Cardiac catheterization showed a subtotal RCA lesion with left-to-right collaterals (Figure 6). His LVEF was severely reduced to 15% to 20% with aneurysm formation. The right heart catheterization showed mildly elevated pulmonary and filling pressures and normal cardiac output. Hypotension was observed during the procedure, and the patient was started on intravenous norepinephrine, and an intra-aortic balloon pump was inserted. Multiple unsuccessful attempts were made to open the RCA occlusion via thrombectomy and balloon angioplasty. Finally, the decision was made to treat the patient medically with a heparin drip and aspirin. After several days in the cardiac intensive care unit and no hemodynamic improvement, the family decided to change the patient's status to comfort care.

## Discussion

More than just microvascular thrombosis

COVID-19 initially appeared to cause more microvascular thrombosis related to pulmonary artery emboli than coronary arterial thrombus. However, clinical experience suggests that in patients with concomitant coronary plaque or other yet undetermined factors, COVID-19 may induce a proclivity to coronary thrombosis. Within the vascular realm, plaque rupture appears to trigger a much more malignant coronary thrombus that is difficult to remove with traditional techniques.

A great imitator: multiple etiologies despite abnormal ECG 

Patients with underlying ischemic heart disease (IHD) have higher mortality from COVID-19 than those without it. However, it also appears that acute myocardial infarction (AMI) has been observed in COVID-19 patients without underlying IHD. Despite the progression of the COVID-19 pandemic, there is still much to learn on how to best manage COVID-19 patients with chronic IHD and those presenting with AMI. Detecting actual AMI in COVID-19 patients based upon ECG changes can sometimes prove challenging as the differential diagnosis may include myocarditis, Takotsubo cardiomyopathy, type 2 AMI, microvascular thrombosis, vasospasm, and typical acute occlusion [3]. Given this broad differential, detailed and strict guidelines have not yet been enacted to manage this patient population. 

Point-of-care ultrasound or limited ECG may help speed the early diagnosis of true heart attack and coronary artery occlusion

Both the American College of Cardiology and the Society for Cardiovascular Angiography and Interventions guidelines agree that in those with a high likelihood of STEMI, a point-of-care ultrasound (POCUS) may have significant utility [4]. Stratification with POCUS for patients with typical symptoms and an ECG consistent with AMI could be valuable. Should POCUS confirm wall motion abnormalities, the STEMI code should be activated, and the patient should be selected for primary percutaneous coronary intervention (PCI). If POCUS and subsequent echocardiography are nonspecific, the clinical judgment of the interventional cardiologist (IC) may determine the best course of action.

Systemic thrombolytic versus mechanical reperfusion with angioplasty or stents in regions where STEMI-receiving hospitals are more than two hours away

The use of systemic fibrinolytics has also been re-evaluated during the COVID-19 pandemic. Their utility was greatly considered when personal protective equipment (PPE) was limited, and mortality rates were higher than they are currently [5]. Among those who come to non-STEMI centers, guidelines similar to those of non-COVID-19 patients apply. The patient should be transferred if PCI can be performed in 120 minutes [6]. Some studies have proposed fibrinolytics as first-line agents in acute coronary syndrome-STEMI [7]. This, however, has not been established as a standard of care unless it is believed that reperfusion time will be delayed. With the decrease in COVID-19-positive patients and the availability of PPE, these patients are more likely to undergo cardiac catheterization according to pre-COVID-19 guidelines rather than being given thrombolytic systemically.

According to these guidelines, PCI remains the standard of care for this subset of patients. However, successful PCI of COVID-19-positive STEMI patients has been challenging. Studies have documented a higher mortality rate in this patient population [5]. Conventional techniques used routinely in PCI during STEMI have not consistently yielded the desired results. This could be due to several factors, including the unique biochemical composition, associated pneumonia and respiratory distress, or the underlying genetic mechanisms. Whatever the etiology, it is imperative to explore other techniques to improve the outcomes in these cases.

Proposals to combat COVID-19 STEMI

In this setting, we propose various methods that a catheterization laboratory may implement to combat COVID-19 STEMI appropriately for a successful PCI. Fundamentally, the catheterization lab should continue to provide adequate and effective PPE to its staff and physicians. Preventing the transmission of COVID-19 is essential.

Mechanical thrombectomy

First, mechanical thrombectomy options should be re-explored. In most COVID-19 STEMI patients, a peripheral thrombectomy device was used to aspirate the thrombus, as the traditional aspiration thrombectomy technique proved inferior. However, even this device has proven to be inadequate. Few cases have evaluated mechanical aspiration techniques in patients with COVID-19 STEMI, with some instances of success. For example, one successful technique described using a stent retriever and aspiration thrombectomy from guide catheter (STRIATE-G) technique. Such a technique involves using a stent retriever and a guiding catheter attached to an aspiration pump that allows for clot retrieval [8]. Newer specialized aspiration devices using neurotracking technology represent some of the most robust systems for mechanical aspiration thrombectomy and should be considered with 29 inHg suction pressure consistently with aspiration. Although not used in routine cases, this combination of a stent retriever and an aspiration pump to aspirate thrombus is effective, especially in embolic myocardial infarction [9]. Some believe that some instances of COVID-19 STEMI may actually evolve from an embolic phenomenon, and thus this approach may be promising [10].

Second, laser thrombectomy should also be considered. Regarding COVID-19 STEMI, this approach has not yet been documented in the literature. However, it has been shown to be effective in other high-risk and highly complex cases. Laser thrombectomy effectively treats neointimal hyperplasia due to re-stenosis of the stent. It also has utility in PCI of degenerated saphenous vein grafts, which often represent complex lesions with high complication rates and high rates of distal embolization [11]. Given the heavy clot burden associated with COVID-19 STEMI patients, a laser might provide enough energy to help dissipate this thick thrombus.

Third, devices such as rotablation and orbital atherectomy, are generally not indicated in acute STEMI with thrombus and are reserved for calcific end-stage plaque; however, in the setting of thrombus, rotablation and orbital atherectomy can also be attempted to restore flow if done carefully and adequately according to the criteria of the injury. One landmark trial showed that the use of orbital atherectomy is relatively safe with a low risk of complications, especially in calcific lesions [12]. Thrombi in patients with COVID-19 STEMI has not necessarily been shown to involve heavily calcified vessels; however, in those with underlying calcific coronary artery disease, the use of these techniques may allow better stent expansion and better outcomes as a result [13]. Compared to this, the recently approved intravascular lithotripsy technique (coronary shockwave) may also be beneficial, although its near-50 atmosphere of shock power is primarily directed at the calcium observed in the arterial media [14].

Optimizing pharmacologic therapies such as P2Y12 inhibition

Traditional pharmacological therapies have included heparin rather than bivalirudin; P2Y12 inhibitors such as clopidogrel, ticagrelor, and prasugrel; and GP2b/3a inhibitors such as tirofiban, eptifibatide, and abciximab. According to Shahzad et al., heparin generally functions as a stronger anticoagulant than bivalirudin in standard dosing and is associated with lower major adverse cardiovascular events with the use of GP2b/3a inhibitors [15]. Based on Wallentin et al. and Schüpke et al., clopidogrel is weaker than ticagrelor [16,17]. According to Mayer et al., ticagrelor is weaker than prasugrel in preventing platelet aggregation [18]. Furthermore, of the GP2b/3a inhibitors, abciximab has the strongest initial dosing with an effective dose within the bolus compared to eptifibatide, whose initial bolus only is approximately 4% of the effective dose [19].

Optimizing standard anticoagulation therapies is a must

It seems essential to optimize traditional standardized therapies for refractory COVID-19-induced coronary thrombus. Activating clotting time (ACT) may need to be dosed higher. If bivalirudin is used, the ACT must be dosed higher, perhaps with additional heparin boluses to the maintenance bivalirudin infusion or increasing bivalirudin dose. If a P2Y12 inhibitor is administered upfront, prasugrel or ticagrelor should be administered as first-line therapy unless contraindicated, such as in the case of a patient weight < 60 kg or an age greater than 75 years (as in prasugrel) [20]. Theoretically, this will not only propagate the effects of P2Y12 inhibition but also decrease platelet aggregation compared to clopidogrel [16]. Unfortunately, abciximab is no longer available in the United States. Its use had been reserved for patients with refractory thrombus or renal failure patients in the past. Effective dosing in the bolus has the potential for benefit in this subgroup of patients.

Consider other pharmaceutical agents, such as intracoronary thrombolytic agents

From anecdotal experience, intracoronary fibrinolytics may also be considered in these patients. According to PCI guidelines, systemic lytic therapy with a GP2b3a inhibitor is usually not indicated due to bleeding concerns. Furthermore, intracoronary thrombolysis is rarely indicated in the modern age of PCI. However, in COVID-19 STEMI patients, these stronger agents may need to be used simultaneously to help decrease the thrombus burden and the overall pro-coagulation environment triggered by the excessive viral load and cytokine storm. Intracoronary thrombolysis can provide a way to concentrate the drug in one area, just as catheter-directed lytic therapy is used in severe pulmonary embolus and ischemic limb disease. This has not yet been effectively documented in this subset of patients; however, it may serve as a last-ditch effort in resistant thrombus, refractory to other more conventional techniques. Intracranial/neurologic contraindications must be carefully weighed versus treatment per prior guidelines.

Decrease infarct size early with cardiac hemodynamic support

Furthermore, it may be crucial to investigate whether early hemodynamic supportive care, either minimally with an intra-aortic balloon pump or more robustly with an Impella device or extracorporeal membrane oxygenation circuit, can decrease the infarcted region in patients with COVID-19 STEMI. When traditional therapy is unsuccessful in the setting of a thrombus, the use of the Impella device may support the patient initially while other antithrombotic therapies are considered. This may potentially lead to a general decrease in infarcted areas and improve patient outcomes (STEMI-Door to Unloading trial ongoing). Early removal of the Impella device can reduce the incidence of associated complications such as ischemia and bleeding. 

## Conclusions

COVID-19 STEMI challenges interventional cardiology as patients encounter life-threatening plaque rupture and coronary artery thrombosis. With extremely high mortality rates, it is imperative to recognize this phenomenon early amongst similar presenting symptoms. POCUS or limited ECG may offer the benefit of early diagnosis. Furthermore, proposed considerations with systemic thrombolysis, mechanical thrombectomy, and anticoagulation agents, such as P2Y12 inhibitors, may prove beneficial in management. With improved technology, better medications to inhibit coagulation and platelet pathways, and optimizing cardiac discharge, ICs may overcome the effects of this SARS-CoV-2 to improve patient survival.
